# Using 360-Degree Video as a Research Stimulus in Digital Health Studies: Lessons Learned

**DOI:** 10.2196/15422

**Published:** 2020-01-06

**Authors:** Brittany A Zulkiewicz, Vanessa Boudewyns, Catherine Gupta, Ari Kirschenbaum, Megan A Lewis

**Affiliations:** 1 Center for Communication Science RTI International Research Triangle Park, NC United States; 2 Multimedia Communication Services RTI International Research Triangle Park, NC United States

**Keywords:** virtual reality, 360-degree video, empathy, migraine disorders, health personnel, medical education

## Abstract

Due to the accessibility of omnidirectional cameras to record 360-degree videos and the technology to view the videos via mobile phones and other devices, 360-degree videos are being used more frequently to place people in different contexts and convey health-related information. Increasingly, 360-degree videos are being employed in health marketing because they have the potential to enhance health-related attitudes and behaviors. As a case study on how this technology may be used for health-related information and its effect on health care providers, we created a 360-degree video that portrays the experience of a migraine sufferer to be used as a stimulus in an online study. We describe the challenges and lessons learned in designing and implementing a 360-degree video as part of an online experiment focused on inducing empathy among clinicians for understanding patient experience. Given the rapid change in digital technology, future research can use this knowledge to design and implement 360-degree video studies more effectively.

## Introduction

An accessible form of virtual reality (VR) technology is 360-degree video. For the public, 360-degree videos are more accessible than other VR technologies because they can be viewed on many common devices, including head-mounted display (HMD) VR headsets, computers, and mobile devices, and because of the technology used to create them. For content creators, 360-degree videos are more accessible because the equipment and labor costs to produce them are lower. For these reasons, 360-degree videos are becoming a more common mode of marketing and communicating information.

To create 360-degree videos, omnidirectional cameras are used to simultaneously record a view from every direction. Users can replicate the way they would change their field of view using their body in the real world by moving the device or clicking the screen. The audience is limited to an omnidirectional view from predetermined standpoints in 360-degree videos, unlike other virtual reality systems that allow users to move throughout a virtual environment. Despite this limitation, 360-degree videos are capable of immersing their audience in a virtual world.

One of the most important capabilities of VR is to transport and fully immerse viewers into a new experience or environment [1]. Similar to traditional narratives and immersive physical environments, immersive technologies permit a sensation of presence. However, immersive technologies may be uniquely capable of helping users feel like they are actually in the environment and experiencing the situation by taking the perspective of the actor. Evidence has shown that immersive technologies can elicit emotional reactions such as empathy [2,3] from users and affect behavior change [4]. Storytelling and reporting using 360-degree video can produce a higher empathic response, immersion, and engagement than text news depicting the same event [5]. In addition, 360-degree video has gained traction in marketing and advertising because it elicits more positive emotion compared with traditional video [6,7]. For example, pharmaceutical companies are starting to use immersive environments in physician-targeted marketing because they may help providers better understand the patient experience. Further, activating empathy through 360-degree video can make the viewer more susceptible to messaging in advertisements that can potentially enhance attitudes and behaviors [8]. Some examples of VR experiences developed by pharmaceutical and medical advertising companies include increasing empathy for migraine sufferers [9] and retinal disease [10].

Because it can place people in unfamiliar environments and experiences, 360-degree video is especially relevant for research focused on understanding how people react to new information or diverse health situations [11]. However, most research is limited to laboratory settings where it is easier to implement the delivery of the video and assess participants reactions. To investigate real-world responses to 360-degree video, an “in the wild” approach is needed to include a larger and more diverse group of people.

This approach can be challenging because of the diverse settings and devices participants may use to view the stimuli [12]. Some researchers purport that HMDs are the most immersive [1]. Several studies have found that HMDs are more effective at eliciting affective responses than a two-dimensional nonimmersive VR interface, personal computer (PC) monitors [13-15]. However, this does not imply that users viewing a 360-degree video on a PC monitor do not feel immersed. One study found that HMD elicited a greater sense of presence than a PC monitor but that the PC monitor was still able to elicit a high sense of presence [13]. Although HMDs may be highly immersive and effective at creating feelings of presence [4], HMDs have limited use in an “in the wild” approach because of costs and availability. Additionally, the novelty of using HMDs can decrease recall [16].

In time, 360-degree videos may become an important medium for studying how different environments and experiences affect behavior and other outcomes. For example, as 360-degree video messaging becomes more ubiquitous, research studies using 360-degree videos as stimuli will be necessary in order to understand how the medium engages viewers and influences their perceptions toward health-related products or influences behaviors among diverse populations. In order to use 360-degree videos as research stimuli in real-world settings, researchers first need to understand how 360-degree videos can be implemented in the real world and how these settings affect proximal outcomes such as immersion, presence, and empathy. To fill this gap, we present lessons learned from an online study examining the effect of a 360-degree video depicting a migraine episode from the sufferer’s point of view on physicians’ empathy compared with a first-person written narrative describing the same experience.

## Methods

We created a 360-degree video that portrays the light and sound sensitivity experienced by migraine sufferers. The content of the video was based on similar videos created by pharmaceutical companies. The video was filmed using an Odyssey (GoPro Inc), a custom camera rig using 16 individual, tethered GoPro cameras designed for Google’s Jump platform. The video was created internally by AK and the John Bollenbacher Multimedia Communication Services department at RTI International.

We fielded the experiment for 3 weeks. Primary care physicians (PCPs) were recruited through an online survey panel vendor, Dynata [17]. Participants included 155 PCPs randomly assigned to 1 of 2 conditions. PCPs assigned to the 360-degree video condition watched a 2-minute 360-degree video portraying what it is like for someone suffering from a migraine at work. PCPs assigned to the narrative condition read a short story about a person’s experience with a migraine at work that mirrored the experience portrayed in the 360-degree video. We then asked all participants to report their feeling of empathy and immersion in the story/video. State empathy was measured using a scale adapted from Shen [18]. The items within the scale were closely correlated in this study, with an alpha of 0.837. To derive the lessons learned, we draw on the results from PCPs assigned to the 360-degree video condition (n=77). The mean age of the 360-degree video condition participants was 49.5 years (SD 10.1, range 30-88), 57% (44/77) were male, and most were non-Hispanic white (45/77, 60%). Below we report on our lessons learned in developing the video and implementing it as part of an online experiment.

## Development

### Virtual Effects

We used After Effects (Adobe), a visual effects and compositing application, to portray the sensory experience of a migraine in a realistic manner. The monoscopic 4k footage was edited in Premiere Pro (Adobe), a video editing application, and then imported into After Effects. In After Effects, multiple stock “steam” elements were composited—layering video clips/instances with transparent background—on top of the espresso machine to represent a subtle increasing stressor to our “barista” neglecting the machine. All light sources in the scene, such as the overhead lights and the sun coming through windows, were masked and given a glow effect, making the light blurry, soft, and expansive to represent the visual sensitivity experienced of a migraine. This was gradually increased throughout the scene. Animated energy effects, such as separate looped video clips, were adjusted to represent white flaring and then composited directly over the already glowing light sources. They were timed to appear midway through the scene. Finally, toward the end of the scene, colorful “coma halo” animations were composited to emanate from the light sources but covered a broader area of the frame to represent a severe visual disturbance for the viewer/barista. The footage was then imported to Premiere for additional color correction, where the entire scene gradually becomes harsher with more contrast and unnatural hues.

### Text Placement

Our 360-degree video starts with a brief text introduction describing the scenario to orient the viewer to the context. Using text in 360-degree video stimuli is challenging because it needs to be placed in a location that is visible from all directions, small enough that the text can be read without changing perspective, and large enough that it can be easily read on mobile device screens. We decided to place the text at 0 and 180 degrees so that part of the text would be visible at any perspective, directing the viewer to change their orientation to view the entire message. We reached this decision after an attempt to place text at more locations in the 360 sphere, which resulted in multiple partial instances of the text being visible by the viewer at one time and proving to be a distraction and an improper introduction to the 360 experience.

### File Size and Format

Although we captured our video at 8k (8192×8192) resolution, viewing platforms such as YouTube and Vimeo do not support such resolutions. Most internet connections and computer processors are also not capable of playing back the content in real time; consequently, 4k was selected as an appropriate and scalable resolution. Also, the file size had to be small enough to support streaming through Wi-Fi and cellular networks and to allow enough resolution to view the text and scenes. The 360-degree video was edited and exported to MP4 files, a common video and audio format supported by popular video platforms such as YouTube and Vimeo.

### Implementation

We successfully conducted an online experiment using 360-degree video as stimuli with very few technical issues. In this section we describe the technical challenges of using 360-degree video in Web surveys and the evidence that we considered when deciding on eligibility criteria based on device type and headphone availability. The results describing the impact of device type and headphone use are shown in Table 1.

**Table 1:** Empathy and engagement for 360-degree video by device type and headphone use.

Item		All (N=77), mean (SD)	Device type		Headphones		
			Desktop/laptop (n=48), mean (SD)	Mobile/tablet (n=29), mean (SD)		No (n=31), mean (SD)	Yes (n=46), mean (SD)
Total time viewing stimuli (seconds)		162.8 (86.2)	171.7 (100.7)	148.0 (52.8)		154.1 (67.7)	175.7 (108.0)
Rating of quality^a^		8.3 (1.6)	8.5 (1.5)	7.9 (1.8)		8.3 (1.6)	8.2 (1.7)
Immersion (I felt immersed in the [video/story].)^b^		3.1 (0.8)	3.3 (0.8)^c^	2.8 (0.8)^c^		3.1 (0.8)	3.1 (0.9)
**Empathy^b^**							
	I could feel the person’s emotions	3.0 (0.9)	3.1 (1.0)	2.8 (0.9)		3.1 (0.7)	2.9 (1.1)
	I can understand what the person was going through	3.2 (0.9)	3.4 (0.8)^c^	2.9 (1.0)^c^		3.3 (0.9)	3.2 (0.9)
	I can relate to what the person was going through	2.9 (1.2)	3.1 (1.1)	2.7 (1.2)		3.0 (1.1)	2.9 (1.2)
	I can identify with the person	2.7 (1.2)	2.7 (1.3)	2.6 (1.1)		2.8 (1.2)	2.6 (1.2)
**Stimuli engagement^d^**							
	The video is memorable	4.1 (0.8)	4.2 (0.7)	3.9 (0.8)		4.1 (0.7)	4.1 (0.8)
	The video is misleading	2.2 (0.7)	2.2 (0.8)	2.3 (0.8)		2.2 (0.8)	2.3 (0.7)
	The video is informative	3.9 (0.7)	4.1 (0.7)^c^	3.6 (0.7)^c^		3.9 (0.6)	3.9 (0.8)
	The video held my attention	4.2 (0.7)	4.3 (0.7)	4.0 (0.8)		4.2 (0.8)	4.2 (0.7)
	I liked the video	3.9 (0.8)	4.0 (0.9)	3.7 (0.7)		3.9 (0.8)	3.9 (0.9)
Watching the 360-degree video changed the way that I think about migraines.^d^		3.5 (0.8)	3.6 (0.8)	3.4 (0.8)		3.3 (0.9)^c^	3.7 (0.8)^c^

^a^Participants rated two quality items on a scale of 1 to 5, with 1=low quality and 5=high quality. These items were summed to create a single quality rating with a range of 2 to 10, with 2=low quality and 10=high quality.

^b^Participants rated items on a scale of 0=not at all to 4=completely.

**^c^**Denotes a significant difference between groups (*P*<.05)

^d^Participants rated items on a scale of 1=strongly disagree to 5=strongly agree.

### Hosting Platform

The 360-degree video technology is relatively new and is not supported by online survey platforms, including the survey platform Qualtrics used in this study. A 360-degree video must be hosted on an outside platform and also be embedded within the survey. We considered five 360-degree video hosting platforms, which are shown in Table 2. We selected OmniVirt Premium because of its hosting capabilities, player customization, and mobile device support. When considering options for hosting and embedding 360-degree video, it is important to consider hosting, player customization, and mobile device support.

**Table 2:** Comparison of 360-degree video Web embedding options as of September 2018.

Platform	Cost	Supported mobile operating systems^a^	Video hosting	Player customization^b^
YouTube	Free	Android	Yes	Limited
Vimeo	$7.99/month	Android	Yes	Full
Google VR View for the Web	Free	Android, iOS	No	Full
OmniVirt (Basic)	Free	Android, iOS	Yes	None
OmniVirt (Premium)	$500/month	Android, iOS	Yes	Full

^a^All options considered support for 360-degree video embedding on all PC operating systems.

^b^Full player customization allows users to remove the logo, disable links to open video on platform, and disable full-screen view for optimal embedding for research.

Videos can be hosted on a private server and embedded using Google VR View or uploaded to an all-in-one hosting and 360-degree video player platform. To save on costs and mitigate technical issues playing the video, we wanted to use a platform that could also host 360-degree video. OmniVirt Premium provided video hosting.

In terms of player customization, many video players allow users to click a logo embedded in the player to view the video on the platform’s website. On some platforms, clicking the full-screen option also opens the video in a new browser window. We needed to disable these options for three reasons. First, opening the video in a new window allows anyone to copy the link and share it with others. Second, participants may not be able to navigate back to the survey to complete the questionnaire. Third, participants would be able to view the video again while completing the survey. OmniVirt Premium allowed us to customize the player to disable the full-screen mode, remove the OmniVirt logo, and disable links.

Regarding mobile device support, many 360-degree video platforms do not support embedded 360-degree video for iOS because of limitations inherent in the Apple iOS operating system. Users must often download the platform app to view the video. We decided to select a platform that supported iOS embedded video because of the popularity of iOS devices. OmniVirt was the only platform that supported Web-embedded 360-degree video playback on iOS devices.

### Connectivity/Stimuli Viewing

Participants can access an online survey using a wide array of devices, browsers, and internet connection speeds. Technical issues or limited bandwidth can cause videos to play choppily or to not play at all. Additionally, participants could potentially skip the video altogether and proceed with the survey. We added several safeguards to ensure that participants watched the video. We disabled the Next button from appearing on the survey screen for 110 seconds, the approximate length of our video. On the same survey screen, we used JavaScript to update a hidden variable each time the play button was clicked.

We also added two questions immediately after the stimuli asking participants to confirm whether they were able to view the 360-degree video, and if they were not able to view it, what technical issues they encountered. All participants who answered that they were not able to view the video were terminated and excluded from analysis. Only one participant was unable to view the video. All participants randomized to the video condition clicked play at least once.

### Device Types

The ability to play 360-degree videos on computers, mobile devices, tablets, and HMD has made 360-degree video more accessible. For research purposes, different device types can confound the results because the different mediums may provide varied levels of immersion and presence because of screen size and video controls. For example, laptop users must click and drag the screen to change perspective, whereas mobile device users only need to change the orientation of their device. Randomized assignment of device type is not an option when conducting online panel research, so eligibility criteria must be limited to participants using a certain device or the device type must be controlled for during analysis.

Prior research has labeled computer monitors as nonimmersive [1] and found that HMD offers a more immersive experience than computers [8,9]. Other research explored the type of viewing platform—such as smartphone with an HMD and smartphone without an HMD—on users’ experiences of immersion and found that adding an HMD to a smartphone did not necessarily lead to more empathy or greater interest in the 360-degree video [3]. Given this, we considered limiting eligibility to mobile device and tablet users. However, because research on the impact of the type of device is equivocal, we opted to include a question asking participants to indicate their device type and explore whether the type of device had a significant effect on any of our key outcomes.

Approximately a third of our final participants (29/77, 38%) viewed the 360-degree video on a tablet or mobile phone, with a majority of participants (48/77, 62%) viewing the video on a laptop or desktop computer. Compared with participants who viewed the video on a tablet or mobile device, participants who viewed the video on a desktop or laptop computer reported significantly greater levels of immersion (mean 3.3 [SD 0.8] vs mean 2.8 [SD 0.8]) and agreement on one dimension of empathy, “I understand what the person in the video is going through” (mean 3.4 [SD 0.8] vs mean 2.9 [SD 1.0]), as shown in Table 1. Choosing not to exclude desktop and laptop users increased the generalizability of our results and improved our participation rate, given the availability of 360-degree video on these devices. Additionally, it appears that viewing the 360-degree video on a desktop or laptop may have been more immersive and impactful than viewing it on a mobile device. Research is needed on the relative immersiveness of mobile devices and laptops and desktops.

### Headphones

Some evidence indicates that using headphones can have an impact on viewers’ immersion in 360-degree videos [19]. We considered adding headphones as a requirement for participation because sound is an integral part of the migraine experience. However, we decided instead to allow users with and without headphones to examine if there was any difference in response to the video. In our instructions, we prompted participants to use headphones or to turn the volume up to 100% if they did not have headphones available. We also asked participants to report whether they had used headphones or not.

A majority of participants used headphones (46/77, 60%). Participants who wore headphones were more likely to agree that watching the 360-degree video changed the way they thought about migraines (mean 3.3 [SD 0.9]) as compared with participants who did not use headphones (mean 3.7 [SD 0.8]; Table 1). We found no significant differences between participants who used headphones and participants who did not use headphones on any other items of engagement, empathy, or immersion. Although there was no association between these outcomes and headphone use, our findings illustrate that a majority of participants had access to headphones and were willing to use them—consequently, including instructions to prompt participants to do so may be advisable—and that wearing headphones may bolster the persuasive power of 360-degree video.

### Conclusion

Given the potential challenges that can arise when creating 360-degree video content and implementing an online experiment using 360-degree video as stimuli, our study found that 360-degree video stimuli must be designed so that essential elements are seen by all viewers, regardless of perspective. The video also must be edited and encoded in a format that allows for playback on common video players and in a resolution that allows participants using a variety of screen sizes to clearly view the scene. The file size of the video must also be small enough to avoid long download or buffering times. Researchers will need to carefully select the survey and video platforms so that all eligible participants can view the stimuli. It also is important to confirm participants actually viewed the stimuli by using embedded variables and follow-up questions. As 360-degree video technology advances, new challenges no doubt will be introduced; however, some of the challenges presented here can be addressed.

Future research also should address the impact of device type and headphone use on empathy and engagement. In this study, we found that laptop and desktop users reported greater levels of empathy and engagement as compared with mobile and tablet device users. Prior research has compared HMD to these types of devices, but no research has looked at the difference between computers and mobile devices, two of most common ways that the general public accesses 360-degree video. Finally, we found that using headphones does not affect most outcomes.

## Abbreviations

HMD: head-mounted display
PC: personal computer
PCP: primary care physician
VR: virtual reality
